# Deciphering the transcriptomic response of *Fusarium verticillioides* in relation to nitrogen availability and the development of sugarcane pokkah boeng disease

**DOI:** 10.1038/srep29692

**Published:** 2016-07-20

**Authors:** Zhenyue Lin, Jihua Wang, Yixue Bao, Qiang Guo, Charles A. Powell, Shiqiang Xu, Baoshan Chen, Muqing Zhang

**Affiliations:** 1 State Key Lab for Conservation and Utilization of Subtropical Agric-Biological Resources, Guangxi University, Nanning, 530005, China; 2 Indian River Research and Education Center, IFAS, University of Florida, Fort Pierce, FL 34945, USA

## Abstract

Pokkah boeng, caused by *Fusarium verticillioides*, is a serious disease in sugarcane industry. The disease severity is related to the sugarcane genotype as well as environmental considerations, such as nitrogen application. The impact of the nitrogen source (ammonium sulfate, urea, or sodium nitrate) on sugarcane pokkah boeng disease and its pathogen was investigated *in planta* and fungal growth and sporulation production was measured *in vitro*. The results showed that ammonium and nitrate were beneficial to fungal mycelium growth, cell densities, and sporulation, which enhanced the disease symptoms of sugarcane pokkah boeng compared to urea fertilization. A total of 1,779 transcripts out of 13,999 annotated genes identified from global transcriptomic analysis were differentially expressed in *F. verticillioides* CNO-1 grown in the different sources of nitrogen. These were found to be involved in nitrogen metabolism, transport, and assimilation. Many of these genes were also associated with pathogenicity based on the PHI-base database. Several transcription factors were found to be associated with specific biological processes related to nitrogen utilization. Our results further demonstrated that nitrogen availability might play an important role in disease development by increasing fungal cell growth as well as influencing the expression of genes required for successful pathogenesis.

Nitrogen availability has been intensively studied in relation to the severity of plant diseases for many years. High nitrogen concentrations frequently increase the magnitude of disease symptoms, which is often attributed to the specific forms of nitrogen available to the pathogen^1^. The biological mineralization of organic nitrogen to inorganic ammonium and its subsequent nitrification to nitrate are dynamic processes resulting in the availability of several forms of nitrogen during plant growth^2^. Thus, the adaptations of pathogens to flourish in these nitrogen-specific environments may be important factors for disease development during successful colonization *in planta*. In order to understand nitrogen utilization in pathogens and to determine which fungal genes are specifically induced under specific nitrogen environments, it is helpful to conduct experiments that include defined nutrient regimens *in vitro*.

Sugarcane is the world’s largest sugar crop and an economically important crop in China. Two *Fusarium* species (*F. verticillioides* gx1 and *F. proliferatum* gx2) have been identified as the causal agents of sugarcane pokkah boeng in China, of which more than 90% are caused by *F. verticillioides*^3^. The symptoms of sugarcane pokkah boeng tend to develop during periods in which high concentrations of nitrogen are applied^4^. Nitrogen availability has significant effects on physiological and morphological characteristics of the fungus, but also on the biosynthesis of secondary metabolites, such as fumonisin in *F. verticillioides*^5^. Fungi are able to respond to quantitative and qualitative changes in nitrogen availability through complex regulatory mechanisms. Genome-wide microarray analyses revealed that the secondary metabolism (SM) gene^6^ expression profiles in *F. fujikuroi* depended on the quantity and quality of the nitrogen source, including the expression of the polyketide synthase (*PKS*) gene cluster^7^. Major GATA transcription factors (*AreA* and *AreB*) and their co-repressor *Nmr* also play a central role in the nitrogen regulatory network^8^. However, despite the progress made in studying nitrogen regulation of secondary metabolism, the molecular action modes as well as possible interactions between the regulators are not well understood. Because of the importance of nitrogen availability in regulating fungal growth, fundamental studies are needed to shed light on the perception of the nitrogen signal and the alteration of downstream gene expression. In the present study, we characterized disease development in *F. verticillioides*-inoculated sugarcane plants exposed to different forms of nitrogen, including sodium nitrate, urea, or ammonium sulfate. We also examined the morphology and growth of *F. verticillioides* CNO-1 and elucidated transcriptome profiles under different nitrogen availability.

## Results

### Disease severity of sugarcane pokkah boeng in response to different forms of nitrogen

**Figure 1:**
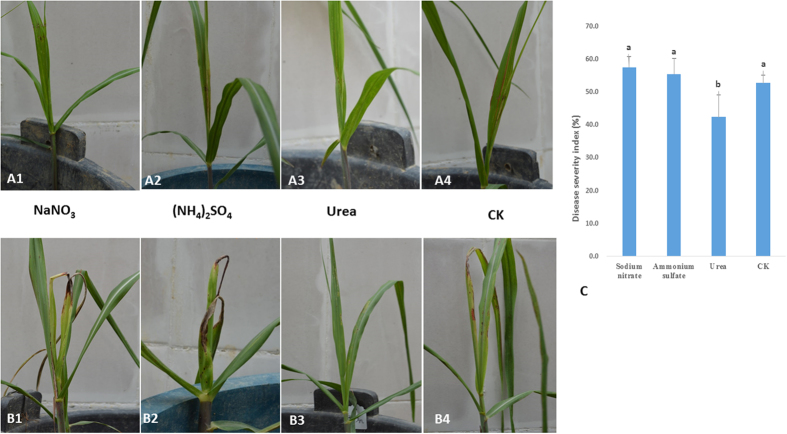
Pokkah boeng disease symptoms (**A**,**B**) and the disease severity index (**C**) % of sugarcane plants fertilized with different kinds of nitrogen and inoculated with *F. verticillioides* CNO-1. Sodium nitrate (**A1**,**B1**) ammonium sulfate (**A2**,**B2**) urea (**A3**,**B3**) and no nitrogen control (**A4**,**B4**). (**A**,**B**) Photos were taken at DAI15 (15 days after inoculation) and DAI30 (30 days after inoculation), respectively. (**C**) Disease severity index (%) in average of sugarcane pokkah boeng disease.

Chlorotic leaves with red stripes of sugarcane pokkah boeng were observed on the control plants (without nitrogen; CK) as well as those fertilized with ammonium sulfate and sodium nitrate, but no red stripes were observed on plants fertilized with urea at fifteen days after initial inoculation (DAI15) (A1, A2, A3, and A4 in Fig. 1). At DAI30, symptoms developed that resulted in leaf death in the control (CK) plants and those treated with ammonium or nitrate, but no significant disease development was observed in the plants fertilized with urea (B1, B2, B3, and B4 in Fig. 1). The disease severity index (DSI) of sugarcane pokkah boeng was significantly lower in the urea-treated plants compared to the control (CK) and the ammonium and nitrate treatment groups (Fig. 1C).

### Characterization of *F. verticillioides* CNO-1 cultured in different nitrogen sources

**Figure 2:**
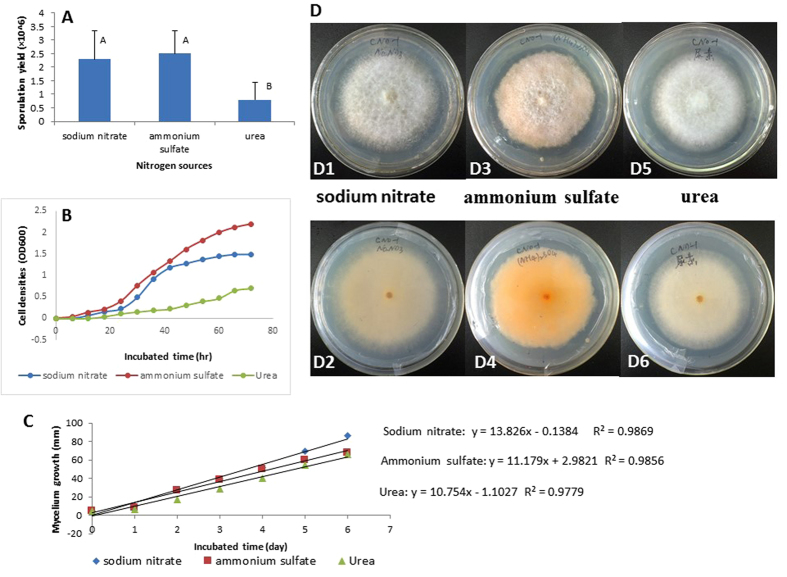
Growth profiles of *F. verticillioides* CNO-1 grown *in vitro* with different sources of nitrogen. (**A)** Sporulation production at DAI 3 (3 days after inoculation); (**B)** Fungal cell densities (OD_600_) at logistic increment; (**C)** Fungal mycelial growth at linear increment; (**D)** Colonies of *F. verticillioides* CNO-1 observed at DAI 3 in sodium nitrate (D_1_, top view; D_2_, bottom view), ammonium sulfate (D_3_, top view; D_4_, bottom view) and urea (D_5_, top view; D_6_, bottom view). Different letters in Fig. 2A indicate highly significant differences (*P* < 0.01). Error bars represent the standard deviation of the mean on at least three replicates.

Phenotypic alterations of *F. verticillioides* CNO-1 were observed in the modified Czapek medium supplemented with different types of nitrogen. CNO-1 showed sparse colony edges when cultured in the urea and nitrate, but colonies were less dense and compact when cultured in the ammonium. Upon aging, colonies developed reddish pigmentation when grown in ammonium, but were white or lightly yellow when grown in urea or nitrate, respectively (Fig. 2D).

CNO-1 grown in different types of nitrogen showed significant differences in the sporulation yield (Fig. 2A), cell densities at logistic increment (Fig. 2B), and mycelium growth at linear increment (Fig. 2C). Higher rates of sporulation and cell densities were observed in the CNO-1 grown in ammonium followed by nitrate, and the lowest rate was observed in urea (Fig. 2B,C), which indicated that *F. verticillioides* grew better in ammonium and nitrate. These observations pointed to a mechanism whereby sugarcane pokkah boeng caused greater disease severity when plants were fertilized with ammonium or nitrate.

### Global transcriptional analysis and identification of differentially expressed genes (DEGs)

Elucidating the set of nitrogen-responsive differentially expressed genes (DEGs) provides insight into how gene expression in CNO-1 responds to different types of nitrogen treatment. Overall, 1,779 (12.7%) genes from a total of 13,999 annotated genes were found to be differentially expressed in CNO-1 treated with at least one type of nitrogen. Based on the complete list of identified DEGs, we constructed a Venn diagram using the Venn diagram package in the R language. Among all of the annotated DEGs, 485 genes were differentially expressed in every comparison between nitrogen treatments (Fig. S1). To understand the transcriptional data in more detail, we also performed hierarchical clustering analysis of the DEGs using Cluster 3.0^9^ with Euclidean distance as the similarity metric and complete linkage as the clustering method. The hierarchical cluster analysis showed a strikingly different and complex pattern of gene expression in response to different kinds of nitrogen (Fig. S2). The largest differences were found in the comparison between CNO-1 treated with ammonium versus urea (Table 1). When compared to nitrate treatment, 608 genes were found to be differentially expressed when fungal cells were grown in urea, including 355 up-regulated and 253 down-regulated DEGs. There were 465 up-regulated and 434 down-regulated DEGs in cells grown in ammonium. However, 664 DEGs were up-regulated and 578 were down-regulated in cells grown in urea compared to those receiving ammonium treatment. These findings indicate that urea treatment induced the up-regulation of a relatively larger number of genes compared to cells grown in either ammonium or nitrate.

### Elucidation of biological functions altered in response to nitrogen availability

To study the *Fusarium* transcriptomic changes resulting from the exposure to urea or ammonium, a GO/KEGG enrichment analysis (P < 0.05) for urea versus nitrate and ammonium versus nitrate was performed, respectively. Briefly, the KEGG term annotation analysis for urea induced the expression of genes involved in several pathways, including protein processing in the endoplasmic reticulum (ko04141), nitrogen metabolism (ko00910), and valine, leucine, and isoleucine degradation (ko00280) (Table 2). These genes could also be categorized by their participation in pathways with functions including the transport of nitrogen, anion, ions, amino acid, organic acid and carboxylic acid (Table S1).

The pathways identified as enriched for ammonium versus nitrate were in the molecular function ontologies ‘oxidoreductase activity’ and ‘oxidation-reduction process’ (Table S1), which are associated with ammonium oxidation for the conversion of ammonia into gaseous nitrogen^12^. In addition, the top ranked categories in the pathway enrichment analysis were butanoate (ko00650), tyrosine (ko00350), and methane (ko00680) metabolism (Table 2), which are related to carbohydrate or amino acid metabolism^13^. There is general agreement that ammonia alone can serve as a source of nitrogen for fungi, the total population achieves the highest growth, and the rate of digestion of carbohydrates is the major factor controlling energy availability for fungal growth and reproduction^10^.

### Nitrogen source affects the pathogen-host interaction

**Figure 3:**
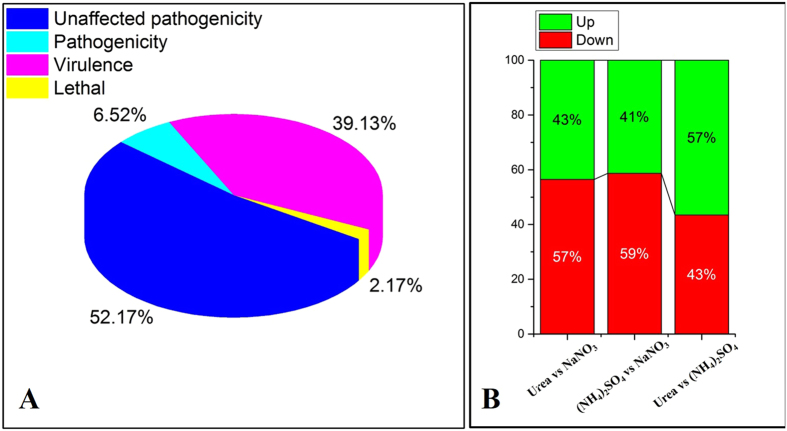
(**A**) Differentially expressed genes related to host-pathogen interaction and its phenotype of pathogen mutant. (**B**) Bar graph presents expression estimates for genes differentially regulated according to nitrogen treatment.

To identify potential virulence- and pathogenicity-associated genes in *F. verticillioides* CNO-1, an all unigenes function in the BLAST analysis (with a cut-off E-value of ≤10^−5^) was conducted against the pathogen-host interaction (PHI) gene database (http://www.phi-base.org), which is a collection of genes shown to affect the outcome of pathogen-host interactions from fungi, oomycetes, and bacteria^11^. After selecting the genes that showed a log2 fold change ≥2 for differential expression in cells grown in different types of nitrogen, we identified 46 putative pathogen-host interaction associated genes (Table S2). A number of *Fusarium* orthologs were found in the cereal pathogenic fungi *F. verticillioides* (3 genes), *F. oxysporum* (1 gene) and *F. graminearum* (2 genes)*, Gibberella zeae* (25 genes), *Magnaporthe oryzae* (4 genes), *Cryptococcus neoformans* (4 genes), *Candida albicans* (2 genes) and others (5 genes). In the search of the PHI database^12^, 24 (52.2%) genes did not affect the fungal pathogenicity, which were assigned to being essential for cellular communication/signal transduction as well as metabolism and transcription between the pathogen and host^13^. The other genes were related to reduced virulence (18 genes; 39.1%), loss of pathogenicity (3 genes; 6.5%), and lethal (1 gene; 2.2%), respectively (Fig. 3A).

Among these 46 genes, the number of genes that were up-regulated increased from 43% to 57% when considering the urea versus nitrate in comparison to the urea versus ammonium (Fig. 3B). The overall variation in the expression of pathogen-host interaction associated genes indicated that their expression was highly influenced by the quality of nitrogen. This observation also helped explain the finding that the disease severity of sugarcane pokkah boeng was affected by the application of different types of nitrogen.

### Cell growth is affected by nitrogen availability

All nitrogen uptake and transformation processes are mediated by enzymatic systems that require carbon, nitrogen, and energy for their synthesis and expression. Through our analyses, we did not detect an abundance of enzymatic pathways responding to different forms of nitrogen, but we did find differential expression of genes involved in nitrogen metabolism (Table S3). The expression of genes for nicotinamide adenine dinucleotide phosphate (*NADP*) or nicotinamide adenine dinucleotide (*NAD*)-dependent glutamate dehydrogenase (*NADP/NAD-GDH*) were highly variable (FDR-corrected P value < 0.01) when cells were grown in ammonium, nitrate, or urea. The highest expression of *NADP/NAD-GDH* was observed in the ammonium treatment, followed by the nitrate and urea treatments. The gene expression levels of glutaminase (*glsA*) and glutamine synthetase (*glnA*) were also highest in the ammonium treatment. The expression of these genes was also validated by qRT-PCR (Fig. S3). These results suggest a key role of glutamate in nitrogen metabolism in CNO-1 and were consistent with previous studies^14^.

The expression of aminomethyltransferase, which catalyzes the release of ammonia and the transfer of a methylene carbon unit to a tetrahydrofolate moiety, was significantly higher in the urea treatment (2.05-fold change in urea versus ammonium), while the transcription of nitrite reductase and nitrate reductase genes (4.78- and 2.85-fold change in nitrate versus urea, respectively) was markedly increased in the nitrate treatment. Table S3 shows that there were also significant differences in the expression of different classes of nitrogen transporters (FDR-corrected P value < 0.01). These biological functions might be important for efficient nitrogen utilization of cells grown on different nitrogen sources.

### Analyses of transcription factors associated with nitrogen availability

We tried to identify several other putative transcription factors that showed significant expression differences in response to different quality of nitrogen. The selection criterion for these nitrogen-dependent regulators was a log2 fold change ≥2 in differential expression between nitrogen treatments. We investigated the potential functions of twelve such putative regulators by finding similar sequences in the Uniprot database (http://www.uniprot.org). Table 3 presents these regulators and estimates of the change in expression of their encoding genes. Interestingly, seven transcription factors seemed to regulate processes related to cell reproduction and phenotypic alterations found in yeast (*e.g.,* regulator of cell meiosis and mitosis, change in cell architecture, abiotic or biotic stress resistance), including *RDR1, SPBP8B7.15c, C15D4, UME6, H3, PNG1,* and *ZFAND1*. Four other transcription factors, including *Acu-15*, oleate-activated transcription factor 1 (*Oaf1*), transcriptional activator of gluconeogenesis *Ert1* and maltose fermentation regulator *Mal13*, were also identified and have been previously shown to be associated with carbon metabolism. These four regulators were differently expressed in cells grown in urea versus ammonium, suggesting that in addition to a role in nitrogen metabolism, nitrogen availability also affected carbon metabolism^15^. Bikaverin is a red pigment in *Fusarium*, and the expression of the bikaverin biosynthetic gene cluster is dependent on the regulator BIK5^7^. BIK5 expression was significantly induced by ammonium treatment, suggesting that the growth of *F. verticillioide* in ammonium increased bikaverin synthesis, which led to the reddish pigmentation *in vitro*.

We also searched for nitrogen-dependent transcription factors previously identified as being involved in nitrogen availability^8^. Unexpectedly, a majority of nitrogen-dependent regulators differed slightly in expression by less than 2-fold from variation in nitrogen source (Table S4, FDR-corrected P value < 0.05, but fold change < 2). For example, eight putative members of the *nit-4* family and five of the *nirA* transcription factor family for nitrogen metabolism were detected in our transcriptional data. *Nit-4*, a member of the *GAL4* family of fungal transcription factors, encodes a protein with an amino-terminal Cys6/Zn2 domain that provides sequence-specific DNA binding^16^. Four of the *nit-4* genes (g2126, g2554, g6634 and g8251) were mildly up-regulated in the nitrate and urea treatments compared to the ammonium treatment, while three genes (g6317, g7312, and g7472) were slightly down-regulated in the urea. *NirA*, nitrogen assimilation transcription factor, activates the transcription of the structural genes for nitrate and nitrite reductases (*niaD* and *niiA*). Two *nirA* genes (g12205 and g5410) were mildly up-regulated in the nitrate and urea, while g11052 was down-regulated in urea. The other nitrogen-dependent transcription factors, including *AreA, AreB, MeaB, Tor,* and *GS*^17^, which associated with secondary metabolite production, mainly had mild shifts in expression levels (<2-fold). Tudzynski previously suggested that the quality or quantity of nitrogen has a unique effect on those regulators, which play important roles in sensing the nitrogen starvation stress and regulate adaptation to new conditions^8^. Herein, we hypothesize that the lack of significant change in the expression of the nitrogen-dependent transcription factors from our dataset may be due to a relatively sufficient supply of the three nitrogen sources.

## Discussion

Nitrogen fertilizers are commonly applied in farming to increase crop yield, but they also modify the ability of several plants to resist pathogen infections^18^. The molecular mechanisms involved in the outcome of the plant-pathogen interactions influenced by nitrogen fertilization is poorly understood. Important factors may include a changing field environment as well as adjustments in gene expression and interaction between the host and the pathogen. Nitrogen availability affects the interaction of the host plant to pathogens in complex regulatory patterns^19^, including (i) the ability to recognize compatible hosts/pathogens; (ii) the ability of hosts to either tolerate the presence of a pathogen or a plant’s susceptibility to pathogens; and (iii) growth or spore production of fungi and the ability of pathogens to colonize host cells. The results presented here showed that nitrogen source (ammonium and nitrate) had a significant influence on the disease severity index of sugarcane pokkah boeng *in planta* as well as the mycelium growth, cell densities, and spore production of its pathogen, *F. verticillioide*, *in vitro*.

### Nitrogen availability and assimilation

**Figure 4:**
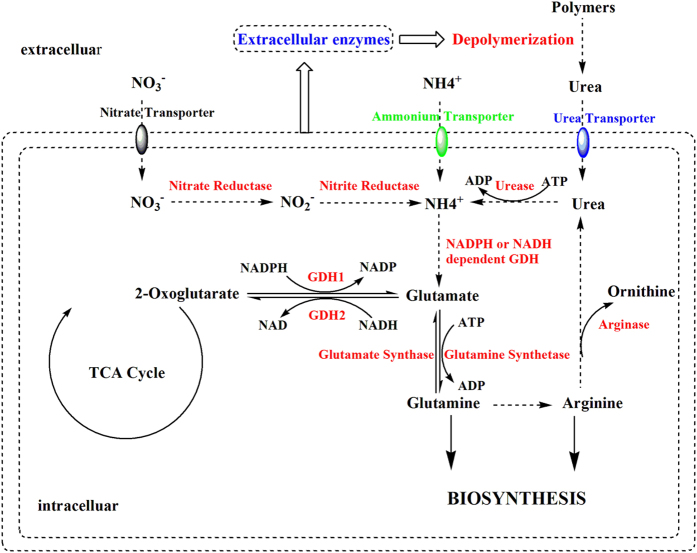
Model of nitrogen assimilation pathway in *F. verticillioides* results in differential expression gene associated to nitrogen metabolism.

Ammonium and nitrate were preferable to mycelium growth, cell density, and sporulation production of *F. verticillioides* CNO-1 and enhanced the disease development of sugarcane pokkah boeng. Fungi have developed different mechanisms for uptake and assimilation of mineral and organic forms of nitrogen, enabling them to utilize a wide range of organic and mineral compounds^20^,^21^. Compared to ammonium, the assimilation of organic polymers in urea requires them first to be broken down to smaller units by extracellular enzymes, which are then taken up directly or degraded further as ammonium^20^. We performed global transcriptome analyses by RNA sequencing to improve our understanding of the *F. verticillioides* response to organic nitrogen source of urea. KEGG/GO enrichment (P < 0.0001) showed a joint up-regulation of ‘general protein biosynthesis and secretion pathways’, ‘transport of nitrogen compound’ and ‘amino acid transmembrane transport’ ontologies in the urea treatment. The transcriptome analyses did indeed indicate that cells that had adapted to nitrogen conditions altered their uptake of nitrogenous compounds and increased the expression of genes encoding high-affinity permeases to maximize the uptake of a limited nitrogen source^15^,^22^. The ontologies ‘oxidation-reduction processes’ and ‘oxidation-reduction activity’ were induced in the ammonium treatment, which might be related to the emergence of ammonium oxidation (*i.e*., converting ammonium into gaseous nitrogen)^23^. By combining the results of the fungal growth characterization and DEGs under three kinds of nitrogen, we found that process networks and pathways associated with nitrogen uptake and assimilation were commonly enriched under different sources of nitrogen (Fig. 4).

Ammonium, which directly assimilates into glutamate, was preferred as a nitrogen source over nitrate and urea for *F. verticillioides* grown *in vitro*. When ammonium was available at high concentrations, the transcription of CNO-1 genes encoding enzymes required for the utilization of nitrate (NO_3_^−^) and organic molecules (urea), such as nitrate transporter, nitrate reductase, nitrite reductase, and arginase genes, were repressed (Table S3). This mechanism is known as nitrogen regulation^24^. However, the up-regulated ammonium transporter 3 member 1 (*AMT1*) of CNO-1 might be capable of highly efficient uptake and transport of NH_4_^+^ across plasma membranes as well as *MepB* participation in ammonium uptake and signal transduction. Once taken up, NH_4_^+^ is predominantly assimilated into biological molecules via either the glutamine synthetase pathway (*GS*) or through glutamate dehydrogenase (*GDH*)^25^. Herein, the highest expression of *GS* and *GDH* was observed distinctly in the ammonium treatment, suggesting that ammonium as a preferable nitrogen source has a higher N assimilation rate. Therefore, ammonium treatment was advantageous for biomass production, mycelium growth, and spore production of CNO-1.

Nitrate can be taken up by specific transporters, but nitrate must be first reduced in two steps by nitrate reductase and nitrite reductase before it can be assimilated in the form of ammonium (Fig. 4). A group of genes (such as nitrate transporter, nitrate reductase, nitrite reductase, and nitrate transporter) involved in nitrate assimilation were up-regulated in the nitrate culture (Table S3). This assimilatory reduction from nitrate to ammonium requires energy^25^. Meanwhile, several regulatory genes, including *nit-4* and *nirA,* had mildly higher expression (fold chang <2) under the nitrate and urea conditions compared to the ammonium condition. These regulatory genes play important roles in controlling the nitrate-induced expression of *niaD* and *niiA*, which encodes nitrate and nitrite reductase at the transcriptional level^16^. This appearance was subject to ammonium repression^26^. Taken together, this is the first evidence of a previously unknown pathway and regulation of nitrate assimilation in *Fusarium*.

Similar to nitrate transporters described in other systems, fungal transport systems for urea are energy dependent. At the early assimilation stages, urease synthesis is activated in the presence of urea^27^, then catalyzed to yield ammonium. Urea may also play a role as a feedback regulator of nitrogen assimilation. The feedback repression of arginase caused by high concentrations of urea^27^ suggested feedback regulation by urea for coordination of nitrogen assimilation processes and a concurrent slowing of cell division. Thus, ammonium assimilates more rapidly and directly than either nitrate or urea^28^. Glutamate dehydrogenase (*GDH*) and glutamine synthetase (*GlnA*) are key enzymes in the fixation of ammonium to amino acids^15^. *GDH* hydrolyzes glutamate to produce 2-oxoglutarate when ammonium is in excess and associated with carbohydrate metabolism^29^. Increased levels of these enzymes may therefore contribute to the higher biomass production of *F. verticillioides* grown in ammonium- or nitrate-supplemented media. In our study, the expressions of *GDH* and *GlnA* were inhibited when urea was provided as the sole nitrogen source.

### Nitrogen sensing and regulation

**Figure 5:**
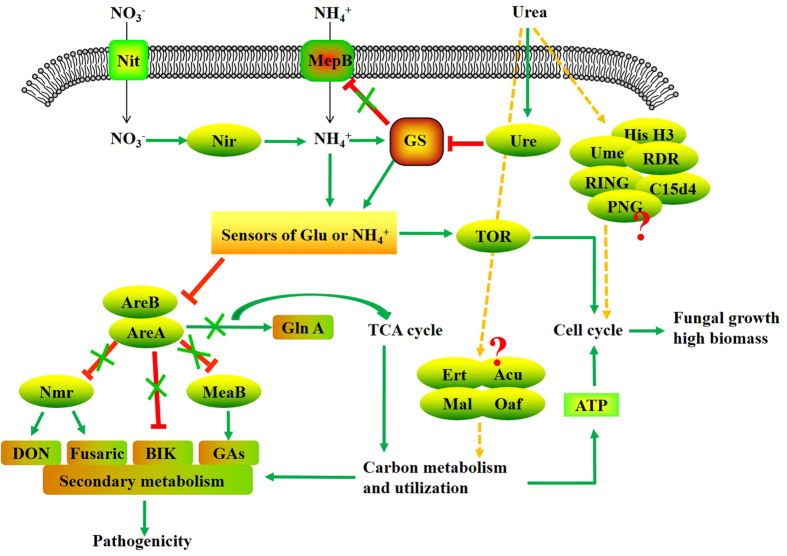
Proposed model of the nitrogen regulation of pathogenicity and biomass production of *F. verticillioides* grown in ammonium-, nitrate- or urea- supplemented media. The nitrogen regulation pathways are charted in the diagram. The known functional pathways are represented as solid lines. The unknown or uncertain functional pathways are represented by dashed lines.

Of the predicted transcription factors involved in nitrogen metabolism, one group was found to be associated with the biosynthesis of secondary metabolites^7^,^8^ (Fig. 5). In our study, several regulators with these described functions were very highly expressed in response to nitrogen source, including *Nit, Nir, Nmr, AreA, MepB, GS, MeaB, Tor,* and *UME6*. Those transcription factors were responsibility for the sensing and coordinated action of pathway global regulatory systems, such as nitrogen and carbon catabolite repression (NCR)^30^. *MepB* is a high-capacity ammonium permease that acts as a nitrogen sensor either by mediating the signal of nitrogen availability or as an intracellular sensor to reduce the intracellular glutamine levels by transducing the glutamine signal to AreA via TOR and/or additional signaling pathways^31^. The conserved *Tor* kinases play a significant role in nutrient sensing and cell growth by affecting nuclear localization of transcription factors, such as *Gln3*, and therefore control genes that are subject to NCR. *GS* plays a regulatory role whereby under ammonium limitation conditions, *GS* binds to *MepB* and blocks the transduction of this repressing signal. This results in the induced expression of *glnA* and likely increases *GS* levels^31^. The GATA factors, *AreA* and *AreB,* together with the co-repressors *NmrA*, *MeaB,* and *BIK*, negatively regulate the regulator of GA, fumonisin, DON, zearalenone, fusarielin H, beauvericin, and arginine catabolism under NCR^30^. In addition, *AreA* is involved in secondary metabolism and is essential for full virulence of plant pathogens (Fig. 5).

**Figure 6:**
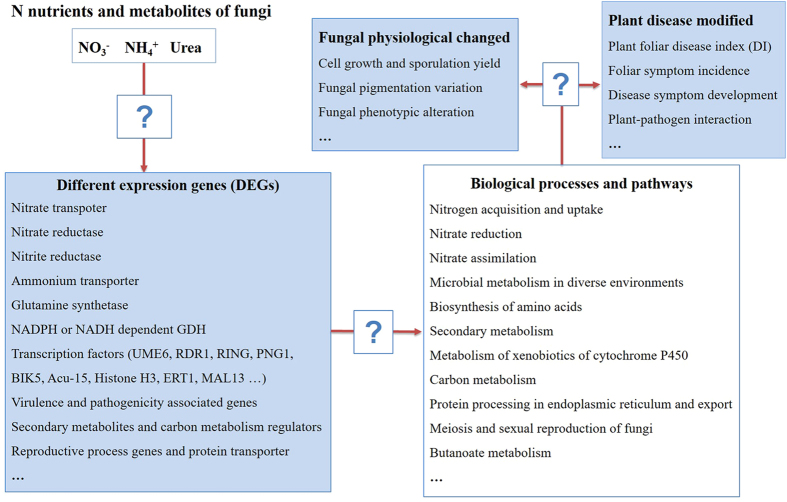
Nitrogen metabolite sensing and signaling results in changes in gene expression and morphological adaptation to nitrogen supply. Boxes with question marks represent molecular mechanisms that are not yet fully understood.

The effects of different nitrogen sources on *F. verticillioides* also resulted in pigmentation variation. Subsequent work revealed that pigment production was associated with fungal secondary metabolites, including the red pigments derived from bikaverin, fusarubins, and carotenoids^7^ as well as the brown and black pigments referred to as melanins^32^. *Bik5* encodes a key regulator in the biosynthetic pathway of the red pigment bikaverin in several fungi^7^. The results presented here showed that ammonium induced abundant expression of *Bik5* in *F. verticillioides*, which was coincident with the appearance of the reddish pigment observed in fungi cultured in ammonium as the sole nitrogen source^33^. Collectively, on one hand, nitrogen excess is sensed by a GS-independent sensor protein, and this signal is transduced by one or multiple putative GS interactor proteins that enhance carbon metabolism and inhibit nitrogen metabolite repression-sensitive gene expression. On the other hand, under nitrogen sufficient conditions, TOR is active and induces the cell cycle, which results in greater biomass. In addition, efficient carbon metabolism is required for the expression of SM cluster genes and fungal growth (Fig. 6).

Other transcripts encoding proteins involved in carbon metabolism showed significant changes (fold change >2) in expression between the urea and ammonium treatments, including *Acu-15, Oaf1, Ert1,* and *Mal13*. All of these were zinc cluster regulators and played central roles in coordinating gene expression during adaptation to different carbon sources^38^. For example, *Acu-15* is involved in regulating the transcription of gluconeogenic genes and *Oaf1* have been shown to mediate the response to oleate by binding as heterodimers to oleate response elements in the promoters of *ß*-oxidation genes; *Ert1* bound the *PCK1* promoter, a key gluconeogenic enzyme; *Mal13* was involved in the control of maltose metabolic genes^34^. Our results suggest that nitrogen availability influenced carbon metabolism by altering transcription rates and/or accumulation and dynamic changes of two aspects of carbon/nitrogen metabolism during fungi growth^35^.

Our study indicated that several putative transcription factors were associated with or responsible for the fungal cell reproduction and phenotypic alterations, including *RDR1, SPBP8B7.15c, C15D4, H3, Bik5, UME6, PNG1,* and *ZFAND1.* These transcription factors exhibited striking differential expression (fold change >2.0) induced by different sources of nitrogen. For instance, *RDR1* genes encoding eukaryotic RNA-dependent RNA polymerases played critical roles in cell developmental regulation, maintenance of genome integrity, and defense against foreign nucleic acids^36^. *SPBP8B7.15c* encodes a protein essential for cell viability and cell size control in *S. cerevisiae*^37^. In addition, the *Ume6* consensus short peptide component element WGCCGCCGA is a key regulator of nitrogen repression and meiotic development^38^. *C15D4* is a key regulator of mitosis and regulates the expression of genes involved in ubiquitin-dependent proteolysis during mitosis^39^. *H3* regulates chromosome dynamics during mitosis and meiosis in *S. cerevisiae*^40^. Moreover, *PNG1*, a conserved ortholog of the fungal kingdom essential protein, is enriched in highly polar cells, such as fungal hyphae^41^. The AN1-type zinc finger protein family is also conserved in fungi and contributes to abiotic stress tolerance, including temperature, salt, and drought^42^. In summary, the DNA or RNA binding properties of transcription factors contribute to fungal pathogenicity and fungal biomass production in very complex networks of pathways that could be affected by different sources of nitrogen.

### Nitrogen relates to fungal pathogenesis

Forty-six genes (Table S2) were putatively associated with host plant-pathogen interactions in *F. verticillioides* cultured under nitrogen conditions. Of these genes, several were associated with reduced virulence (18; 39.1%), loss of pathogenicity (3; 6.52%), and lethality (1; 2.17%), respectively (Fig. 4A). Included among this set of 46 genes were orthologs of *CLC-A, MGV1, CLA4,CaAft2, nlpI, CpkA, chs2, LEU2, CPA1, HDF3, GAS2, Cak1,* and *GzZC089*. For example, ammonium induced an approximately 5-fold increase in the expression of *CLA-4*, which is essential for virulence and morphological switching of *Candida albicans*^43^. Nitrate induced the expression of *MGV1*, which is involved in multiple developmental processes related to sexual reproduction, plant infection, and cell wall integrity in *F. graminearum*^44^. Urea up-regulated the expression of *CaAft2,* which plays an important role in iron metabolism and is associated with *C. albicans* pathogenicity and virulence^45^. However, 24 genes (52.1%) that are not required for pathogenicity but were previous associated with pathogen-host interactions^7^. *STE12* (alpha) is a protein kinase involved in pathogenesis that contributes to the capsule size of the yeast cells and is important for the expression of several virulence-associated genes^46^. *Bcpme2* is an important fungal pectin lytic enzyme involved in plant invasion, since pectin is a major component of the primary cell wall and middle lamella and most fungal pathogens must degrade it when encountering the physical barriers of a plant cell^47^. *FVEG_2520* is predicted to encode a hydrolase that plays a role in fumaric acid synthesis and the production of mycotoxins^48^. These results indicate that nitrogen fertilization influences the outcome of the *F. verticillioides* CNO-1 interaction with sugarcane using a variety of mechanisms, including changes in pathogenicity and virulence.

The relationship between nitrogen availability and fungal pathogenesis is complex and variable. Studies presented here emphasized the role of ammonia and nitrate as important aspects in *Fusarium* pathogenesis. Results of the transcriptome and fungal growth and reproduction analysis indicated the effects of ammonium and nitrate comparison to urea were preferential for the growth of *F. verticillioides*. The number of up-regulated DEGs associated with host plant-pathogen interactions increased from 43% to 57% when considering the urea versus nitrate in comparison to the urea versus ammonium, suggesting that *F. verticillioides* strongly responds to ammonium through plant-pathogen interaction genes. These results support the hypothesis that ammonium and nitrate fertilization appear to enhance the disease symptoms of sugarcane pokkah boeng.

In this study, a comprehensive network of gene expression in *F. verticillioides* was established to analyze processes modulated by different sources of nitrogen and to identify new regulatory mechanisms. However, many aspects of these molecular mechanisms are not yet fully understood (Fig. 6), including (i) how nitrogen metabolites are sensed, and (ii) the unidentified signal transduction pathways downstream of the nitrogen signal, which result in global transcriptional changes. Future studies are required to pinpoint commonalities in the involvement of nitrogen in the induction of pathogenicity and physiological factors of *Fusarium*^33^. Previous studies have implicated roles for miRNA-regulated networks, protein-protein interactions, protein to DNA and miRNA to RNA^49^, and hormone regulatory sub-networks in the fungal response to nitrogen availability^8^,^28^,^50^. We envision that integrative systems biology in combination with genomics approaches will play a more prominent role in generating integrated dynamic models of *Fusarium* responses to nitrogen in the future.

## Materials and Methods

### Assays of sugarcane pokkah boeng disease

Two-bud seed cane cuttings of sugarcane (sugarcane hybrid, ROC22) were treated with carbendazim at a concentration of 100 ppm for 30 min., four of which were planted in a 10-liter pot filled with a mixture (1:1, vol/vol) of vermiculite and pozzolana (inert crushed volcanic rock) on March, 2015. The seedlings were irrigated with sterile water for one month, then they were micro-injected with 100 μl of conidial suspension (10^7^ CFU.ml^−1^) of *F. verticillioides* CNO-1 using a sterile injector. The inoculated plants were grown in an insect-proof greenhouse at 28 °C with a photoperiod of 16 h light: 8 h dark^51^. A randomized complete block design with three replicates was carried out on the inoculated sugarcane plants fertilized using different forms of nitrogen (sodium nitrate, ammonium sulfate or urea) at the nitrogen concentration of 45 mM. No nitrogen treatment was used as a control.

Each plot were assessed individually based on symptoms on all leaves and scored using a symptom severity scale ranging from 0 to 4:0, no symptom; 1, only one leaf symptom, chlorotic with or without red stripes, wrinkling and splitting of the leaves; 2, more than two leaf symptoms, ladder-like shaped lesions on the spindle leaves, pronounced yellowing, wrinkling of spindle, twisting or tangling appearance of spindle and red stripes; 3, top rot of spindle leaves, pronounced red stripes with white spore mass; 4, leaf death. Disease severity index (DSI) of each replicate was calculated as DSI = 100 × (Σ score/4 × *N*), with *N* being the number of sugarcane seedlings observed (*N* = 20). A two-way ANOVA statistical analyses were performed using SAS 9.3 software (SAS Institute Inc.).

### Culturing of *F. verticillioide*s CNO-1

*F. verticillioides* CNO-1 is the pathogen of sugarcane pokkah boeng isolated from Guangxi, China, in 2012. The isolate was kept on a potato dextrose agar (PDA) slant medium at 4 °C until used. The spores of *F. verticillioides* CNO-1 (1 mL, 1.0 × 10^6^ conidia/mL) were grown in modified Czapek medium (containing per L: K_2_HPO_4_ 1 g, MgSO_4_·7H_2_O 0.5 g, KCl 0.5 g, Fe_2_SO_4_ 0.01 g, sucrose 30 g, and nitrogen at the same total concentration of 45 mM of sodium nitrate, ammonium sulfate or urea, respectively). The flasks were shaken on a rotary shaker at 200 rpm and 28 °C for 6 days. Fungal growth was measured in triplicate by determining optical density at 600 nm (OD_600_) with a Spectronic 21 D spectrophotometer (Milton Roy, Chicago, IL). The cell suspension spore concentrations were determined at day 3 after inoculation (DAI3) by direct counting using a hemocytometer^10^. Mycelium growth was measured on agar plates. Mycelia of CNO-1 growing in the three forms of nitrogen were harvested with three biological replicates. The harvested mycelia for immediate total RNA extraction were centrifuged at 5,000 × g for 15 min at 4 °C.

### RNA extraction, cDNA synthesis and RNA sequencing

RNAs was extracted using the TRIzol^®^ Reagent RNA Isolation Kit (Invitrogen, Grand Island, NY) following the manufacturer’s protocol from three biological replicates of each treatment resulting in 9 samples. The mRNA was purified using Dynabeads^®^ mRNA Purification Kit (Invitrogen, Grand Island, NY). RNA degradation and contamination was detected on 1.2% agarose gels. RNA sequencing libraries were created using an Illumina TruSeq^TM^ RNA sample prep kit (Illumina, SD, USA) following the standard high-throughput protocol. Sequencing was conducted on an Illumina HiSeq2500 instrument at Shanghai Majorbio Bio-pharm Technology Co., Ltd (Shanghai, China). By removing adaptor sequences, empty reads and low quality reads using SeqPrep (https://github.com/jstjohn/SeqPrep), the resulting quality of the thinned reads was visualized using FastQC^60^, only those terminal bases with a Phred quality score under 30 were trimmed. Cleaned reads of a length threshold of 200 bp were mapped onto the reference genome of *F. verticillioides* strain 7600 (http://www.ncbi.nlm.nih.gov/assembly/GCA_000149555.1/) using TopHat 1.2.0 (http://tophat.cbcb.umd.edu/)^52^,^53^. Phrap Clustering tools were used to obtain sequences that could not be extended at either end (a minimum of 95% identity, a minimum of 35 overlapping bases, a minimum of 35 scores, and a maximum of 20 unmatched overhanging bases at sequence ends)^54^. The obtained sequences were defined as unigenes. The repetitive genes were identified in the final gene set by BLAST search against genome sequences and identification of those genes with two or more matches to the genome with at least 50% coverage or an E-value less than 10^−30^.

### Transcriptome assembly, annotation, gene ontology and metabolic pathway analysis

The generated unigenes were compared with the NCBI non-redundant (nr) database using the BLASTx algorithm (http://www.ncbi.nlm.nih.gov/), with a cut-off E-value of ≤10^−5^. Gene Ontology (GO) (http://www.geneontology.org/) annotations and the corresponding enzyme commission numbers (EC) of the sequences were obtained using Blast2go software (http://www. blast2go.org/). Perl scripts were created to extract the metabolic annotation data from KEGG (Kyoto Encyclopedia of Genes and Genomes) metabolic annotation files (ftp://ftp.genome.jp/pub/kegg/genes/organisms/pic/).

### Identification and functional enrichment analysis of the differentially expressed genes (DEGs)

Gene expression levels were measured as fragment per kilo base per million reads (FPKM) as described by Mortazaviet *et al*.^55^. For RNA-seq based expression analysis, FPKM for each defined gene was calculated by Cufflinks and Cuffdiff (http://cufflinks.cbcb.umd.edu/)^56^. For these analyses, genes with extremely low expression in all libraries (FPKM less than 0.1) were excluded.

The differentially expressed genes, their corresponding attributes, fold changes (FC) (in log2 scale), p-values, and q-values (false discovery rate corrected p values) were reported in the output files from Cuffdiff. The significance of the gene expression difference was determined as yes if the p-value was less than the false discovery rate (FDR) after Benjamini-Hochberg correction for multiple testing^57^. FDR < 0.05 and |logFC|>2 were set as the threshold for significant differential expression. The statistical significance of the functional GO Slim enrichment was evaluated using the Fisher’s exact test within Blast2GO (FDR < 0.05)^58^. Significantly enriched KEGG pathways were identified with KOBAS 2.0^59^ using a hypergeometric test and the Benjamini-Hochberg FDR correction.

### Quantitative RT-PCR validation of target genes

Each RNA sample was adjusted to 1 μg/μl with nuclease-free water. Two μg of total RNA was reverse transcribed in a 20 μl reaction volume using the Prime Script^TM^ RT reagent Kit with gDNA Eraser (Takara, Dalian, China). The *F. verticillioides* ACT1 (actin) gene was used as an internal control. The qRT-PCR was performed using the SYBR^®^ *Premix Ex Taq*™ (Tli-RNase H Plus) (Takara, Dalian, China), according to the manufacturer protocol. A no template control (NTC) (nuclease-free water) was included in the experiment to detect contamination and to determine the degree of dimer formation. The qRT-PCR analysis was performed in triplicate for each extracted RNA sample. PCR amplifications were performed using a Roche Light Cycler 480 (Indianapolis, IN, USA) in a 20 μl reaction volume. The data were analyzed using Light Cycler^®^480 software. A relative quantitative method (^ΔΔ^Ct) was used to evaluate the quantitative variation^60^. The sequences of the primer sets used are detailed in Table S5.

## Additional Information

**How to cite this article**: Lin, Z. *et al*. Deciphering the transcriptomic response of *Fusarium verticillioides* in relation to nitrogen availability and the development of sugarcane pokkah boeng disease. *Sci. Rep.* **6**, 29692; doi: 10.1038/srep29692 (2016).

## Supplementary Material

Supplementary Figures

Supplementary Tables

## Figures and Tables

**Table 1:** Summary of differentially expressed genes.

	Urea *vs* NaNO_3_	(NH_4_)_2_SO_4_ *vs* NaNO_3_	Urea *vs* (NH_4_)_2_SO_4_
Total	608	899	1242
Up	355	465	664
Down	253	434	578

**Table 2:** The results of KEGG pathway enrichment analysis (P < 0.05).

ID	Term	Count	P-Value
Urea *vs* NaNO_3_			
ko04141	Protein processing in endoplasmic reticulum	10	0.000411939
ko00910	Nitrogen metabolism	6	0.000456639
ko00280	Valine, leucine and isoleucine degradation	8	0.000515465
ko00480	Glutathione metabolism	6	0.002863017
ko00564	Glycerophospholipid metabolism	5	0.013846186
ko00980	Metabolism of xenobiotics by cytochrome P450	4	0.028111916
ko00982	Drug metabolism - cytochrome P450	4	0.033870658
ko00130	Ubiquinone and other terpenoid-quinone biosynthesis	2	0.044170098
ko04122	Sulfur relay system	2	0.044170098
(NH_4_)_2_SO_4_ *vs* NaNO_**3**_			
ko00650	Butanoate metabolism	13	9.64E–05
ko00350	Tyrosine metabolism	13	0.001462676
ko00680	Methane metabolism	7	0.012878017
ko00980	Metabolism of xenobiotics by cytochrome P450	6	0.014210688
ko00950	Isoquinoline alkaloid biosynthesis	6	0.01631289
ko00930	Caprolactam degradation	4	0.023873186
ko00740	Riboflavin metabolism	5	0.02698433
ko04960	Aldosterone-regulated sodium reabsorption	2	0.031651075
ko00410	beta-Alanine metabolism	6	0.041147201
ko00380	Tryptophan metabolism	9	0.042086439
ko00280	Valine, leucine and isoleucine degradation	7	0.044141472
ko00643	Styrene degradation	4	0.045233341
Urea *vs* (NH_4_)_**2**_SO4			
ko00280	Valine, leucine and isoleucine degradation	12	0.000826494
ko00650	Butanoate metabolism	13	0.001208374
ko00630	Glyoxylate and dicarboxylate metabolism	6	0.012740128
ko00410	beta-Alanine metabolism	8	0.015958219
ko00680	Methane metabolism	8	0.015958219
ko04978	Mineral absorption	3	0.019333684
ko00643	Styrene degradation	5	0.028740599
ko00640	Propanoate metabolism	8	0.029623986
ko00380	Tryptophan metabolism	11	0.037025952
ko00360	Phenylalanine metabolism	8	0.049754516

**Table 3:** The list of the potential nitrogen-dependent transcription factors (log2 fold change > = 2, FDR–corrected *P* value < 0.05).

Gene ID	Description	log2		
		Urea *vs*NaNO_3_	(NH_4_)_2_SO_4_ *vs* NaNO_3_	Urea *vs* (NH_4_)_2_SO_4_
g11073	Protein RDR1 (*RDR1*)	1.625	3.819^a^	−2.194^a^
g12844	RING finger protein P8B7.15c (*SPBP8B7.15c*)	−0.323	−2.040^a^	1.717
g13343	Transcriptional regulatory protein UME6 (*UME6*)	0.268	−2.035^a^	2.303^a^
g8408	Bikaverin cluster-transcription factor (*Bik5*)	−0.374	3.713^a^	−4.088^a^
g11282	Transcriptional regulatory protein C15D4 (*C15D4*)	−2.148^a^	1.731	1.596
g6352	Protein PNG1 (*PNG1*)	−2.179^a^	−1.664	−0.514
g8663	Transcriptional activator protein Acu-15 (*Acu-15*)	−2.096^a^	−0.095	−2.001^a^
g8689	Histone H3 (*H3*)	−2.076^a^	−0.295	−1.781
g11758	Oleate-activated transcription factor 1 (*Oaf1*)	0.993	−1.643	2.636^a^
g12386	Transcription activator of gluconeogenesis Eet1 (*Eet1*)	0.783	−1.756	2.539^a^
g1854	AN1-type zinc finger protein 1 (*ZFAND1*)	−0.929	1.66	−2.588^a^
g686	Maltose fermentation regulatory protein Mal13 (*Mal13*)	0.396	−1.853	2.250^a^

^a^Indicates significance (log2| > = 2).
