# Supplementary material for: Deciphering the transcriptomic response of Fusarium verticillioides in relation to nitrogen availability and the development of sugarcane pokkah boeng disease
Source: Sci Rep. 2016 Jul 20;6:29692. doi: 10.1038/srep29692 (PMC4951700; doi:10.1038/srep29692)
Supplement: Supplementary Figures [file srep29692-s1.pdf]

Supplementary Figure S1-S4

Deciphering the transcriptomic response of Fusarium verticillioides in relation to nitrogen availability and the development of sugarcane pokkah boeng disease

Zhenyue Lin[1ξ], Jihua Wang[1ξ], Yixue Bao[1ξ], Qiang Guo[1], Charles A. Powell[2], Shiqiang Xu[1], Baoshan Chen[1], Muqing Zhang[1,2*]

[1] State Key Lab for Conservation and Utilization of Subtropical Agric-Biological Resources, Guangxi University, Nanning, 530005, China

[2] Indian River Research and Education Center, IFAS, University of Florida, Fort Pierce, FL 34945, USA

[ξ] These authors contributed equally to this work.

* Correspondence and requests for materials should be addressed to

Dr. Muqing Zhang (mqzhang@ufl.edu)

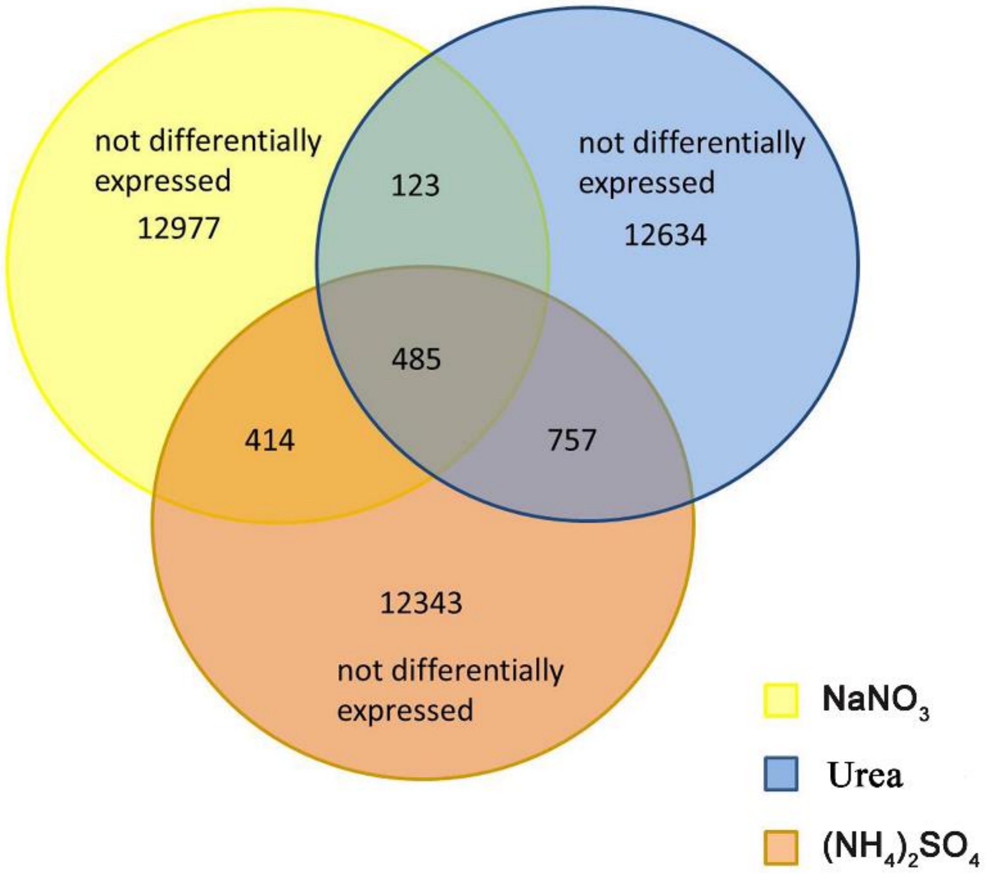

Fig S1. Venn diagrams representing differential expression of genes whose expression was detected by RNA-seq. The number of differentially expressed genes in each condition as well as the overlap between the different conditions is presented.

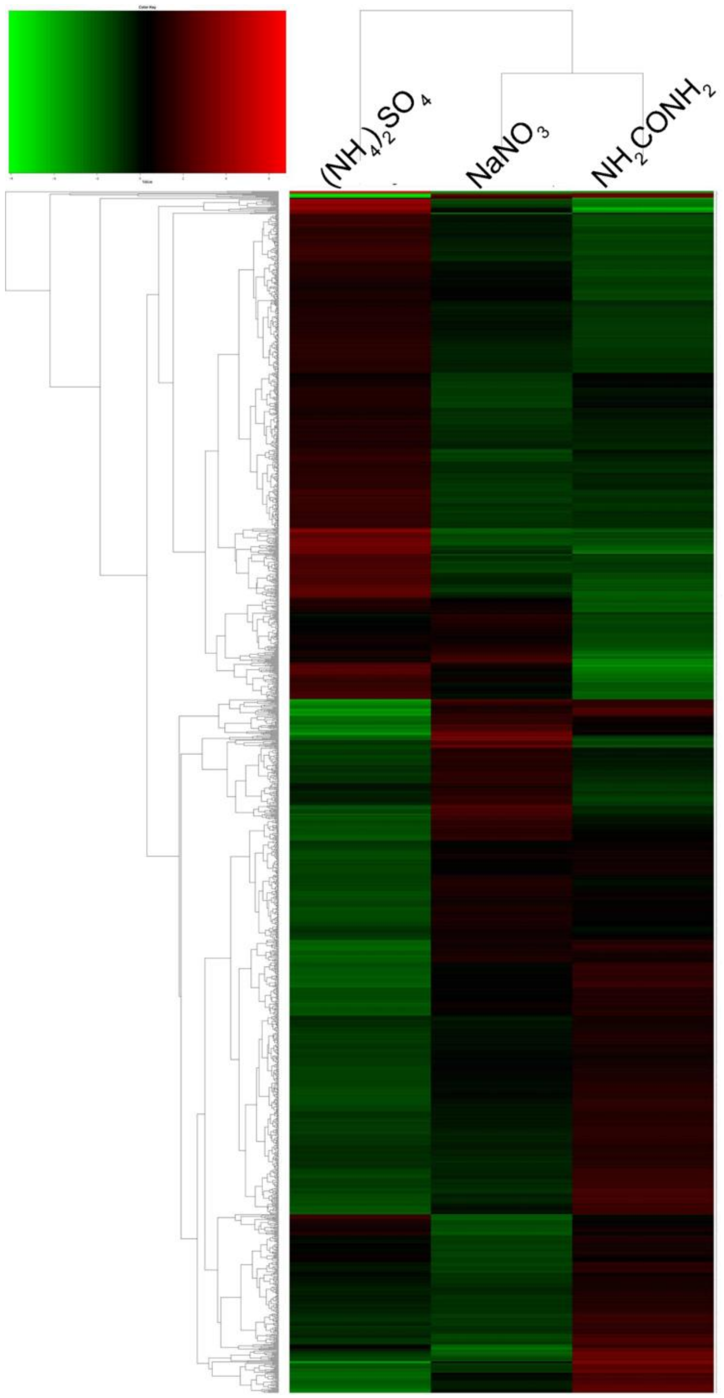

Fig S2. Hierarchical clustering analysis of gene expression. The dendrogram at the top of the figure indicates relatedness of the three samples based on overall gene expression values. The dendrogram on the left side of the figure orders genes into groups based on the divergence of expression values among the three conditions. The colors indicate relative levels of gene expression: red indicates a higher gene expression level, green indicates lower expression and black indicates the median value. This figure was generated using a log scale of RPKM values.

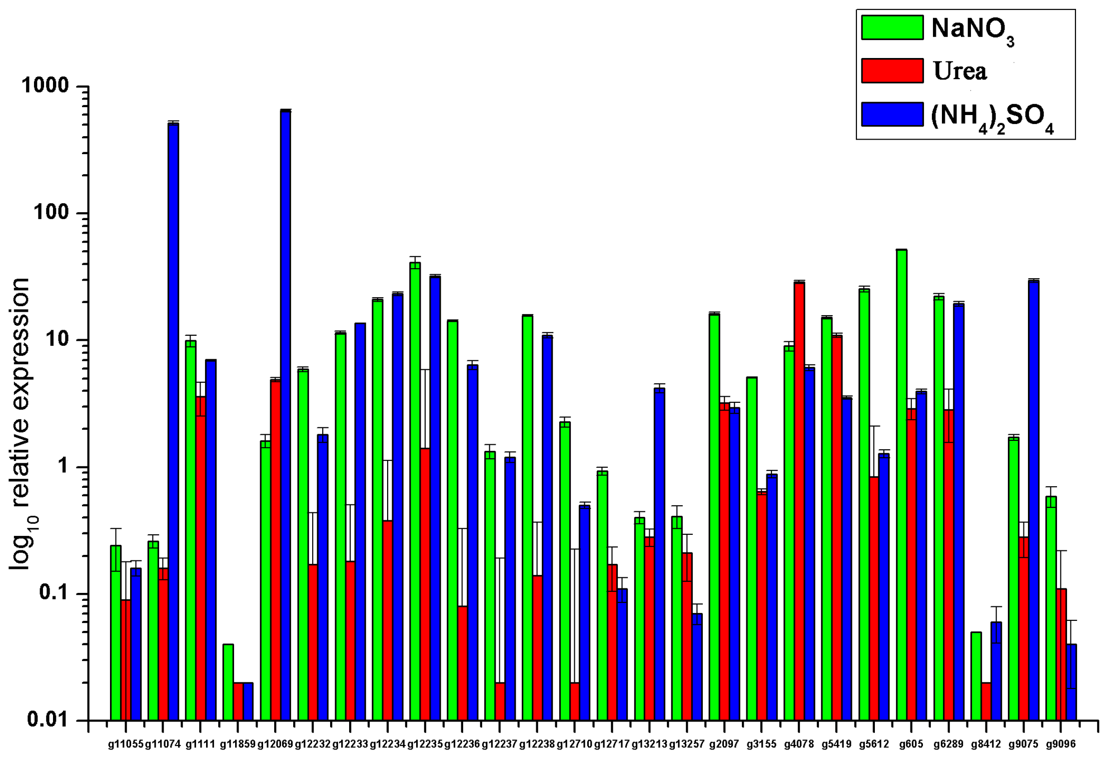

Fig S3. qRT-PCR analysis of selected genes.

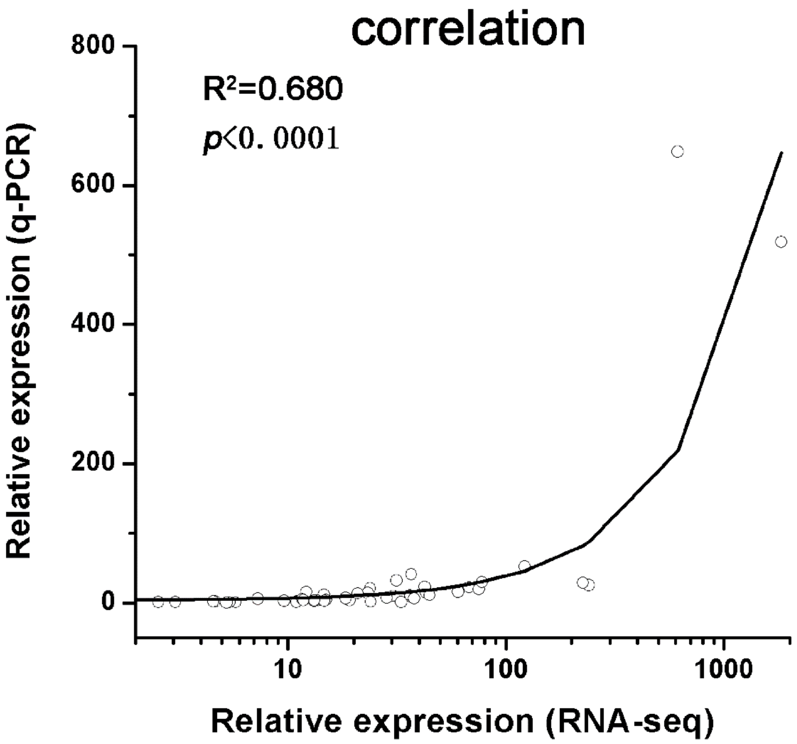

Fig S4. Correlation between qRT-PCR and RNA-seq. Regression t-tests were conducted to determine the significance of the correlation.
