# Supplementary material for: Deciphering the transcriptomic response of Fusarium verticillioides in relation to nitrogen availability and the development of sugarcane pokkah boeng disease
Source: Sci Rep. 2016 Jul 20;6:29692. doi: 10.1038/srep29692 (PMC4951700; doi:10.1038/srep29692)
Supplement: Supplementary Tables [file srep29692-s2.pdf]

Supplementary Table S1-S5

Deciphering the transcriptomic response of Fusarium verticillioides in relation to nitrogen availability and the development of sugarcane pokkah boeng disease

Zhenyue Lin[1ξ], Jihua Wang[1ξ], Yixue Bao[1ξ], Qiang Guo[1], Charles A. Powell[2], Shiqiang Xu[1], Baoshan Chen[1], Muqing Zhang[1,2*]

[1] State Key Lab for Conservation and Utilization of Subtropical Agric-Biological Resources, Guangxi University, Nanning, 530005, China

[2] Indian River Research and Education Center, IFAS, University of Florida, Fort Pierce, FL 34945, USA

[ξ] These authors contributed equally to this work.

* Correspondence and requests for materials should be addressed to

Dr. Muqing Zhang (mqzhang@ufl.edu)

Table S1. Results of GO pathway enrichment analysis.

ID	GO description	Ratio in DEGs	P-Value	Type
**Urea vs $NaNO_3$**				
GO:0071705	nitrogen compound transport	24/608	5.43E-07	biological process
GO:0008509	anion transmembrane transporter activity	21/608	2.92E-06	molecular function
GO:0006820	anion transport	22/608	5.01E-06	biological process
GO:0003333	amino acid transmembrane transport	17/608	1.21E-05	biological process
GO:0015171	amino acid transmembrane transporter activity	17/608	1.86E-05	molecular function
GO:0006865	amino acid transport	17/608	2.82E-05	biological process
GO:0005342	organic acid transmembrane transporter activity	17/608	4.21E-05	molecular function
GO:0008514	organic anion transmembrane transporter activity	17/608	4.21E-05	molecular function
GO:0046943	carboxylic acid transmembrane transporter activity	17/608	4.21E-05	molecular function
GO:0015849	organic acid transport	17/608	7.48E-05	biological process
GO:0046942	carboxylic acid transport	17/608	7.48E-05	biological process
GO:0034220	ion transmembrane transport	21/608	0.000173	biological process
GO:0015711	organic anion transport	17/608	0.000218	biological process
GO:0015075	ion transmembrane transporter activity	30/608	0.000505	molecular function
GO:0006811	ion transport	31/608	0.00231	biological process
GO:0016491	oxidoreductase activity	99/608	0.0063	molecular function
GO:0022891	substrate-specific transmembrane transporter activity	37/608	0.0149	molecular function
GO:0055114	oxidation-reduction process	91/608	0.0329	biological process
GO:0022892	substrate-specific transporter activity	38/608	0.0352	molecular function
GO:0022857	transmembrane transporter activity	41/608	0.120	molecular function
GO:0020037	heme binding	21/608	0.148	molecular function
GO:0046906	tetrapyrrole binding	21/608	0.148	molecular function
GO:0005215	transporter activity	44/608	0.628	molecular function

**$(NH_4)_2SO_4$ vs $NaNO_3$**				
GO:0055114	oxidation-reduction process	136/899	0.000179	biological process
GO:0016491	oxidoreductase activity	141/899	0.000558	molecular function
GO:0042537	benzene-containing compound metabolic process	9/899	0.0668	biological process
GO:0003824	catalytic activity	352/899	0.553	molecular function
GO:0042542	response to hydrogen peroxide	4/899	0.609	biological process
GO:0018966	styrene metabolic process	4/899	0.609	biological process
GO:0070301	cellular response to hydrogen peroxide	4/899	0.609	biological process
GO:0042207	styrene catabolic process	4/899	0.609	biological process
GO:0042744	hydrogen peroxide catabolic process	4/899	0.609	biological process
GO:0044282	small molecule catabolic process	16/899	0.649	biological process
GO:0044712	single-organism catabolic process	16/899	0.649	biological process
GO:0043446	cellular alkane metabolic process	5/899	0.705	biological process
GO:0015947	methane metabolic process	5/899	0.705	biological process
GO:0009063	cellular amino acid catabolic process	14/899	0.712	biological process
GO:0015075	ion transmembrane transporter activity	31/899	0.728	molecular function
GO:0046395	carboxylic acid catabolic process	15/899	0.774	biological process
GO:0016054	organic acid catabolic process	15/899	0.774	biological process
GO:0006804	peroxidase reaction	8/899	0.890	biological process
GO:0004601	peroxidase activity	8/899	0.890	molecular function
GO:0016684	oxidoreductase activity, acting on peroxide as acceptor	8/899	0.890	molecular function
**Urea vs $(NH_4)_2SO_4$**				
GO:0016491	oxidoreductase activity	189/1242	6.28E-05	molecular function
GO:0055114	oxidation-reduction process	178/1242	0.000106	biological process
GO:0019482	beta-alanine metabolic process	5/1242	0.138	biological process
GO:0003824	catalytic activity	474/1242	0.852	molecular function

Table S2. Lists of genes related to pathogen-host interaction and different expression.

Gnen ID	Gene name	Blast hit	Pathogen species	Phenotype of mutant	Similarity (%)	e-value	log2		
							Urea vs $NaNO_3$	$(NH_4)_2SO_4$ vs $NaNO_3$	Urea vs (NH4)2SO4
g1308	bcpme2	PHI:1029	*Botrytis cinerea*	Unaffected pathogenicity	90.04	0	0.828	2.624 [a]	-1.795
g7904	CLA4	PHI:85	*Candida albicans*	Reduced virulence	92.66	0	-0.327	5.686 [a]	-6.013 [a]
g10135	CaAFT2	PHI:2555	*Candida albicans*	Reduced virulence	83.69	0	2.057 [a]	-0.269	2.325 [a]
g3968	CLC-A	PHI:286	*Cryptococcus neoformans*	Reduced virulence	99.23	0	0.518	3.056 [a]	-2.538 [a]
g4940	CPA1	PHI:213	*Cryptococcus neoformans*	Reduced virulence	86.01	0	-2.18 [a]	-2.222	0.041
g3707	LAC2	PHI:397	*Cryptococcus neoformans*	Unaffected pathogenicity	79.51	0	-2.927 [a]	1.109	-4.037 [a]
g12767	STE12alpha	PHI:156	*Cryptococcus neoformans*	Unaffected pathogenicity	77.14	0	-2.499 [a]	3.362 [a]	-5.862 [a]
g3700	nlpI	PHI:2459	*Erwinia amylovora*	Reduced virulence	92.59	0	-0.228	2.006 [a]	-2.233 [a]
g10173	MGV1	PHI:266	*Fusarium graminearum*	Loss of pathogenicity	94.96	0	-0.973	-4.977	4.004 [a]
g6245	FGSG_04610	PHI:1094	*Fusarium graminearum*	Unaffected pathogenicity	80.58	2.00E-53	-0.644	1.364	-2.009 [a]
g7492	FOXG_00076	PHI:1101	*Fusarium oxysporum*	Unaffected pathogenicity	88.14	5.00E-157	2.904 [a]	-2.348 [a]	5.252 [a]
g9002	FVEG_12531	PHI:3383	*Fusarium verticillioides*	Unaffected pathogenicity	98.15	0	1.247	-1.134	2.381 [a]
g8995	FVEG_12520	PHI:3390	*Fusarium verticillioides*	Unaffected pathogenicity	95.88	0	0.943	-1.329	2.271 [a]
g8996	FVEG_12519	PHI:3391	*Fusarium verticillioides*	Unaffected pathogenicity	91.25	0	1.517	-1.342	2.859 [a]
g8025	Sc Cdc28	PHI:1178	*Gibberella zeae*	Reduced virulence	97.22	0	1.591	-0.839	2.429 [a]
g159	GzC2H019	PHI:1359	*Gibberella zeae*	Unaffected pathogenicity	96.51	2.00E-126	-0.678	1.364	-2.042
g7213	GzC2H055	PHI:1392	*Gibberella zeae*	Unaffected pathogenicity	92.31	0	2.156 [a]	1.242	0.913

g7104	GzbZIP007	PHI:1325	*Gibberella zeae*	Reduced virulence	92.11	5.00E-103	-1.315	0.862	-2.178 [a]
g2437	GzWing018	PHI:1646	*Gibberella zeae*	Reduced virulence	91.77	0	0.61	-2.843 [a]	3.453 [a]
g5530	GzbHLH008	PHI:1307	*Gibberella zeae*	Unaffected pathogenicity	90.38	0	-1.457	-2.703 [a]	1.246
g7226	FgVELB	PHI:2427	*Gibberella zeae*	Reduced virulence	89.15	0	-1.551	0.791	-2.342 [a]
g1843	GzZC041	PHI:1726	*Gibberella zeae*	Unaffected pathogenicity	88.35	0	-1.015	1.581	-2.596 [a]
g12844	GzAra003	PHI:1455	*Gibberella zeae*	Unaffected pathogenicity	86.65	0	-0.323	-2.04 [a]	1.717
g8663	GzMyb004	PHI:1540	*Gibberella zeae*	Unaffected pathogenicity	86.39	0	-2.096 [a]	-0.095	-2.001 [a]
g4383	GzbHLH011	PHI:1310	*Gibberella zeae*	Unaffected pathogenicity	85.49	0	-2.021 [a]	-1.306	-0.716
g6921	GzZC023	PHI:1708	*Gibberella zeae*	Unaffected pathogenicity	85.37	2.00E-117	2.177 [a]	1.13	1.047
g6139	HDF3	PHI:1174	*Gibberella zeae*	Reduced virulence	83.41	0	0.612	-2.463 [a]	3.075 [a]
g8050	GzbHLH004	PHI:1303	*Gibberella zeae*	Unaffected pathogenicity	83.23	0	0.089	-2.058 [a]	2.147 [a]
g11111	GzZC127	PHI:1812	*Gibberella zeae*	Unaffected pathogenicity	82.95	8.00E-166	-2.275 [a]	-0.108	-2.167 [a]
g1515	GzHMG013	PHI:1481	*Gibberella zeae*	Unaffected pathogenicity	81.15	0	-1.333	-2.97	1.637
g10483	Sc Cak1	PHI:1192	*Gibberella zeae*	Reduced virulence	79.14	0	2.039 [a]	1.322	0.717
g1728	GzZC077	PHI:1762	*Gibberella zeae*	Unaffected pathogenicity	79.08	0	2.382 [a]	0.454	1.928
g8027	GzHMG002	PHI:1470	*Gibberella zeae*	Reduced virulence	78.98	4.00E-68	0.28	-1.877	2.157 [a]
g3942	GzZC183	PHI:1868	*Gibberella zeae*	Unaffected pathogenicity	78.71	0	-2.036 [a]	-1.247	-0.789
g8027	GzHMG002	PHI:1470	*Gibberella zeae*	Reduced virulence	77.37	3.00E-157	0.28	-1.877	2.157 [a]
g11592	GzZC089	PHI:1774	*Gibberella zeae*	Lethal	77.14	0	-0.202	-2.612 [a]	2.41 [a]
g12386	GzMyb003	PHI:1539	*Gibberella zeae*	Unaffected pathogenicity	76.22	0	0.783	-1.756	2.539 [a]
g10340	GzHMG037	PHI:1505	*Gibberella zeae*	Unaffected pathogenicity	76.12	0	2.167 [a]	-1.59	3.757 [a]
g535	GzZC194	PHI:1879	*Gibberella zeae*	Unaffected pathogenicity	75.28	0	-1.323	1.232	-2.556 [a]
g13610	MGG_06188	PHI:2169	*Magnaporthe oryzae*	Reduced virulence	81.82	1.00E-167	0.412	2.638 [a]	-2.227 [a]
g5484	ATG8	PHI:2061	*Magnaporthe oryzae*	Loss of pathogenicity	99.44	0	-1.855	-2.266 [a]	0.411
g12848	GAS2	PHI:257	*Magnaporthe oryzae*	Reduced virulence	79.85	0	-0.97	1.57	-2.54 [a]
g9951	MoPEX7	PHI:2155	*Magnaporthe oryzae*	Loss of pathogenicity	89.32	1.00E-157	-0.789	-2.277 [a]	1.488

g6234	LEU2	PHI:504	*Saccharomyces cerevisiae*	Reduced virulence	86.54	0	-0.418	-2.212 [a]	1.794
g4792	CpkA	PHI:1082	*Stagonospora nodorum*	Reduced virulence	91.61	0	-2.426 [a]	-1.277	-1.149
g277	chs2	PHI:1109	*Ustilago maydis*	Reduced virulence	89.04	2.00E-118	-1.084	1.142	-2.226 [a]

[a] Indicates significant (log2|>=2).

Table S3. *F. verticillioides* nutrition-related genes and transcripts expressed during the different sole nitrogen source culture condition. Signaling components are not included.

Gene ID	Gene symbol	Description	Relative expression level (RPKM)		
			$NaNO_3$	Urea	$(NH_4)_2SO_4$
g9306	glnA	Glutamine synthetase	2151.740	1022.240	2271.83
g8908	-	Putative urea active transporter 1	27.421	0.259	3.749
g8674	E6.3.5.4, asnB	Putative asparagine synthetase	151.281	231.470	205.373
g8367	-	Putative urea active transporter 1	0	0.038	0.080
g8287	E3.5.1.1, ansA, ansB	Putative L-asparaginase 3	0.092	0.513	0
g7588	GDH2	Glutamate dehydrogenase (NAD-dependent)	31.835	21.540	55.358
g6235	URE	Urease	12.393	13.618	18.219
g4204	-	Putative urea carboxylase	1.461	1.201	1.294
g605	narK, narU, nasA	Nitrate transporter	121.776	9.629	11.814
g5612	E1.7.1.3, niaD	Nitrate reductase	240.085	33.110	23.967
g5149	E1.4.1.4, gdhA	NADP-specific glutamate dehydrogenase	206.618	10.681	280.398
g497	-	Ammonium transporter MEP3	208.606	3.460	61.682
g4832	E3.5.3.1, rocF, arg	Arginase	11.122	7.568	1.177
g4734	E3.5.3.1, rocF, arg	Arginase	239.280	363.568	257.631
g4078	E1.13.12.16	Nitronate monooxygenase	36.318	225.521	38.008
g3419	E1.7.1.4L, nirB	Nitrite reductase	742.756	26.982	22.841
g228	gcvT, AMT	Putative aminomethyltransferase	373.895	601.078	146.015
g13741	glsA, GLS	Glutaminase	16.425	36.697	45.673
g13726	TC.AMT	Ammonium transporter 3 member 1	0.390	0.259	31.816
g11981	-	Ammonium transporter 1	0.073	0.232	0.000
g11552	GLT1	Putative glutamate synthase [NADPH]	41.435	49.761	72.026

g11173	E3.5.3.1, rocF, arg	Arginase	3.237	3.137	0.480
g10508	-	Nitrate reductase	0	0.166	0
g10366	glnA	Glutamine synthetase 2	98.127	77.029	41.722

Table S4. Nitrogen-dependent transcription factors and differentially expressed genes encoding these transcription factors.

Transcription factors and its regulation	Gene ID	Relative expression level (RPKM)		
		$NaNO_3$	Urea	$(NH_4)_2SO_4$
nit-4 (a pathway-specific regulatory gene, controls the nitrate-induced expression of niaD and *niiA*, which encode nitrate and nitrite reductase at the transcriptional level, respectively)	g12089	0.000	0.000	0.027
	g2126	3.957	1.519	0.155
	g2554	23.720	12.778	0.636
	g6317	3.349	6.383	6.852
	g6634	33.705	34.463	19.946
	g7321	2.320	5.233	4.493
	g7472	1.124	3.085	3.637
	g8251	30.901	19.390	9.875
nirA (an activator of transcription initiation of *niaD* and *niiA*)	g11052	5.059	1.994	6.322
	g12205	3.836	5.778	2.367
	g2379	5.865	6.065	6.403
	g5410	13.582	26.854	9.236
AreA (a positively acting GATA family of transcriptional regulators comprising a DNA-binding domain of the C2C2 Zinc finger type)	g3393	53.664	67.746	29.700
	g13393	0.000	0.000	0.533
	g2021	52.968	40.160	33.244
	g5531	69.139	50.970	48.972
	g7214	255.014	132.919	118.613
	g6205	286.155	194.978	79.353
Nmr (a negative-acting regulatory gene, binding AreA in the presence of glutamine and thus inhibit AreA activity)	g3064	0.112	0.807	0.280
	g5180	86.049	61.891	142.989

MepB (Ammonium Permeases, participation in ammonium uptake and signal transduction)	g1661	0.629	1.294	1.958
	g1885	18.781	30.710	20.383
	g6972	38.870	31.246	26.886
GS (Glutamine synthetase, involving in ribosome biogenesis and translation initiation)	g9306	2151.740	1022.240	2271.830
	g10366	98.127	77.029	41.722
MeaB (the transcription factor, coordinately mediate nitrogen repression with AreA)	g6102	190.032	128.322	93.591
Tor (target of rapamycin, essential for viability and regulate translation initiation and cell cycle progression)	g3228	942.067	780.217	457.751

Table S5. Sequences of primers used for qRT-PCR

Gene ID	Description	Forward	Reverse
g11055	3-hydroxybenzoate 6-hydroxylase	ATCCGCCATGACTACTCTACT	GCAGTGTCCTTGAGGTGATTAT
g11074	hypothetical protein FOXB_08591	CACCATTTACCAAAGGCAGTTC	GAACTTGGTGCGAAAGCATATT
g1111	Meiotically up-regulated protein 69 protein	GCTCGCTCTATCTGGCTTTA	CGTGTAGTTGGGTCGTACTT
g11859	Fcyp (Similar to Conidial yellow pigment)	CCGGGTCTGTTAGTGTTGTT	GACAATGATGCTGGCCAAAG
g12069	hypothetical protein FOXB_02953	CCTACACTGGTATCGACAAGC	CAAGACCAGCAGCCATAGT
g12232	cytochrome P450 monooxygenase [C13 oxidase]	TCCTGCCAATGTCTGTCTTTC	GGTGGTGATGTGGGTGTATTT
g12233	copalyl-diphosphate/kaurene synthetase	CCACCGACAAGATCATAGACAG	GCTGAGTGACTCGTGATGATAG
g12234	geranylgeranyl diphosphate synthase	AGCGACCTTCTCATCAACATC	CAGTCTAAGCTCCCGTTTGTT
g12235	cytochrome P450 monooxygenase [GA20 oxidase]	CATGAAGCCTGGTAGCATAGT	GGGATCATCAAGGCGTCTATT
g12236	GA14-synthase	CTCATGGACAGCGTCTTGAA	GTGAGTTTGTTCTTGGGTAGGA
g12237	Ent-kaurene oxidase=Cytochrome P450-4	GTCTCGCGTCTATTCACACTAC	GCCTTTGACCTCGTCTCTAAG
g12238	GA4 desaturase	GTGCTACTGACCAAGGCTAATC	GCTATATCACTCCCGTCCTTCT
g12710	PKS 18	GTCGAGCAGTCAGGCTATTT	TCGTAGTCAGCAGCACAAG
g12717	FSP1	CCGCCTATTCGAGGTGTTATC	GAGTCCTGCAAGGTGTTAGTAG
g13213	oxidoreductase	GTGTTGTTCGACTTCCTCCTAC	CATATGCAGAGACGCCCTTT
g13257	related to acid phosphatase precursor	CTTAAGTCTGGGAAGGCTCATC	CTCCGCCAACCAGTGATATT
g2097	Peroxisomal catalase	GGAAGAGATGCTCGGTTAGTATG	TACCCTCATCAGTCCTCATCTT
g3155	probable chitosanase precursor	TCGACTGTGATGGAGCAAATAA	TGGCATCCAAATCAGGGATAC
g4078	Nitronate monooxygenase	CCTGCCTCTTATAGCCAAGTATC	CATTGCCAACCTGGACAAAC
g5419	ATP-dependent DNA helicase II subunit 1	CGTCTAGAGAGCGGTCTGATAA	AGGTCATCCTGGAATGGTAGAG
g5612	nitrate reductase	GGATCCCAACAGAGGTGTTATG	TTGCCCAGGAATGACGATAC
g605	Nitrate transporter	GAGTCATTGCCGACATCATCTA	TTTCCGATGACAATGAGGAGAG
g6289	Meiotically up-regulated protein 14	CATGCCATGCTCACTCTAAGTA	GCTGTGGTCGTTGTAGAAGAT

g8412	PKS 4	GCTTTGGAGGAGAACCTGAA	GAGCAGTAGTCGTGTACGAAAG
g9075	Catalase	TACCCTGTTTGGAAGCTGTATG	GGAAACTCCTTGTGAGACCAA
g9096	Lambda-crystallin like protein	GTTAGCTGAGGCCGACTTTAT	GCCGAGCATGTTTGTCTAATG
Reference gene	related actin gene	GAGAACGAGCGTGTCTTGATTGAGCC	TTTCCTCCGCAGAATGAAGAAGGACTC
